# Physical properties of carbon nanowalls synthesized by the ICP-PECVD method vs. the growth time

**DOI:** 10.1038/s41598-021-97997-8

**Published:** 2021-09-29

**Authors:** Yerassyl Yerlanuly, Rakhymzhan Zhumadilov, Renata Nemkayeva, Berik Uzakbaiuly, Almaz R. Beisenbayev, Zhumabay Bakenov, Tlekkabul Ramazanov, Maratbek Gabdullin, Annie Ng, Viktor V. Brus, Askhat N. Jumabekov

**Affiliations:** 1 Laboratory of Engineering Profile, Al-Farabi Kazakh National University, 050040 Almaty, Kazakhstan; 2 Kazakh-British Technical University, 050000 Almaty, Kazakhstan; 3 Department of Physics, Nazarbayev University, 010000 Nur-Sultan, Kazakhstan; 4 National Nanotechnology Laboratory Open Type, Al-Farabi Kazakh National University, 050040 Almaty, Kazakhstan; 5 National Laboratory Astana, 010000 Nur-Sultan, Kazakhstan; 6 Department of Chemical and Materials Engineering, Nazarbayev University, 010000 Nur-Sultan, Kazakhstan; 7 Department of Electrical and Computer Engineering, Nazarbayev University, 010000 Nur-Sultan, Kazakhstan

**Keywords:** Materials for devices, Electronic devices, Sensors and biosensors, Materials for energy and catalysis, Batteries, Fuel cells, Porous materials, Solar cells, Thermoelectrics, Nanoscale materials, Graphene, Structural properties, Synthesis and processing, Two-dimensional materials

## Abstract

Investigation of the physical properties of carbon nanowall (CNW) films is carried out in correlation with the growth time. The structural, electronic, optical and electrical properties of CNW films are investigated using electron microscopy, Raman spectroscopy, X-ray photoelectron spectroscopy, ultraviolet photoelectron spectroscopy, UV–Vis spectroscopy, Hall Effect measurement system, Four Point Probing system, and thermoelectric measurements. Shorter growth time results in thinner CNW films with a densely spaced labyrinth structure, while a longer growth time results in thicker CNW films with a petal structure. These changes in morphology further lead to changes in the structural, optical, and electrical properties of the CNW.

## Introduction

Carbon nanowalls (CNWs) are one of the allotropic modifications of carbon and are three-dimensional networks of vertically oriented graphene sheets^1,2^. CNWs can be synthesized on various metallic, semiconductor and insulator substrates^3^ using various techniques^4^. Carbon atoms in CNWs form covalent chemical bonds with sp^3^ (C–C) and sp^2^ (C=C) hybridization, which afford this unique nanostructured material to demonstrate unique structural, morphological, electrical, optical and chemical properties^1,2,5,6^. In recent years, CNWs have found many applications in various electronic devices such as solar cells^7–10^, light-emitting diodes^7–12^, and sensors^13,14^. For instance, Lin et al.^9^ showed that CNWs grown on quartz substrates have good conductivity and tunable optical transmittance (from 90.4 to 67.8% at 550 nm). The perovskite solar cells fabricated on substrates with a CNW film, in which the CNW layer is used as the charge collecting layer, achieved reasonably high-power conversion efficiencies (PCEs) close to 7% and enhanced device stability^9^. This demonstrates a potential use of CNW films in manufacturing of cost-effective transparent conducting electrodes for various optoelectronic applications. Moreover, a high specific surface area and high electrical conductivity of CNWs make them attractive for electrochemical studies and supercapacitor applications^15–17^. For instance, a composite of CNWs and ZnO possesses high Faraday capacitance and electrical conductivity^18,19^. Guerra et al. have reported that electrodes made of CNWs coated with ZnO possess 23 times higher surface capacitance compared to the ones with CNWs alone^20^. In addition, excellent mechanical properties and super hydrophobic nature of CNWs make this material extremely attractive for wearable electronics applications^21,22^ and designing water-repellent coatings^23,24^.

Despite a wide range of applications, controlling the synthesis process and the final film morphology of CNWs remain challenging, in particular, obtaining CNWs with required morphology and material properties^25–29^. In this regard, studying the effect of various synthesis parameters on the morphology of the CNW films has an important research value^30–33^.Varying synthesis parameters leads to significant changes in morphology of CNWs, which subsequently alters the physical and chemical properties of the obtained material^34^. Gaining a better understanding of the relationship between the structural and electro-optical properties would make it extremely useful to obtain CNWs with desired properties for further practical applications. With this in mind, in this work the effect of material growth time on the physical properties of CNW films is systematically studied. The investigated CNW films are synthesized using inductively coupled plasma enhanced chemical vapor deposition (ICP-PECVD) method. In this contribution, we revealed in details the coupling between structural, electronic, optical, and electrical properties of the obtained CNW films with the material growth time during the ICP-PECVD process. It is demonstrated that material growth time has a significant effect on the morphology of CNW films. Shorter growth time results in smaller thickness CNW films with a densely arranged maze-like structure, whereas longer growth time results in larger thickness CNW films with a petal-like structure. Generally, the obtained CNW films are semitransparent and behave more like a semiconductor material.

## Experimental part

CNW films on quartz substrates are synthesized using the ICP-PECVD method, employing a PECVD Split Tube Furnace system (OTF-1200X-PEC4LV, MTI). The experimental setup consists of a CVD furnace with a 76 mm diameter quartz tube and an inductive coil connected to a high-frequency generator (13.56 MHz) with an automatic matching device. A gas supply system is connected to one side of the quartz tube, whereas the other side is connected to a roughing vacuum pump. The experiments are carried out as follows. In the first stage, quartz substrates (1 × 1 cm) are placed into the quartz tube, and the working zone is sealed. Then, the quartz tube is evacuated up to 10^–2^ Torr. Afterward, the substrates are heated up to 800 °C. At this point, the vacuum tube is filled with argon gas at the flow rate of 5 sccm, and induction plasma is ignited at 140 W. The quartz substrates are maintained under these conditions for 10 min in order to remove residual contaminants from the surface of the substrates and to allow formation of hotspots. Following the plasma treatment stage, a mixture of argon (Ar) and methane (CH_4_) gas (89.1% Ar and 9.9% CH_4_) and hydrogen (H_2_) gas are introduced into the quartz tube. The flow rates for Ar/CH_4_ mixture and H_2_ gases are 20 sccm and 5 sccm, respectively. Here, H_2_ is used for decomposition of CH_4_ and as a reaction source for the nucleation process. The carbon atoms and radicals, which are obtained from decomposition of the CH_4_, are absorbed by the hotspots formed during the pretreatment stage. This leads to the formation of graphite nano-islands, which then yield vertically-oriented few-layer graphene sheets. These graphene sheets eventually grow into a continuous CNW film with various morphologies depending on the growth time^28,29,35^. In the experiments, the growth time for CNW films is varied from 30 to 60 min with a 10 min step.

Morphology of the obtained CNW films is characterized using a scanning electron microscope (SEM, ZEISS Crossbeam 540), whereas a transmission electron microscope (TEM, JEOL JEM-1400 Plus) and a Raman spectrometer (LabRAM Horiba Evolution & Omega Scope with the laser wavelength of 514.5 nm) are used to investigate the structural properties of the samples. Electronic properties of the CNW films are characterized using an X-ray photoelectron spectrometer (XPS) with a monochromatic X-ray source Al-Kα radiation at 1486.6 eV (NEXSA, Thermo Scientific) and an ultraviolet photoelectron spectrometer (UPS, NEXSA, Thermo Scientific). The optical properties of the samples are investigated using a UV–Vis spectrometer (Lambda 1050, PerkinElmer Ltd.), whereas a Van-der-Pauw Hall effect measurement system (HMS-5500, Ecopia) and a four-point probe measurement system (RM3000, Jandel) are used to test the electrical properties of the obtained CNW films. Silver paste electrodes were deposited on the CNW thin films for electrical and thermoelectrical measurements. The Seebeck coefficient $S$ was determined by linear fitting of experimentally measured thermal voltage ${V}_{t}$ at different temperature gradients between the contacts $S=\frac{\Delta {V}_{t}}{\Delta T}$. The temperature difference was controlled by two thermocouples attached to the sample. The thermoelectric setup was calibrated by measuring indium tin oxide thin films on glass substrates.

## Results and discussion

### Morphological and structural properties

**Figure 1:**
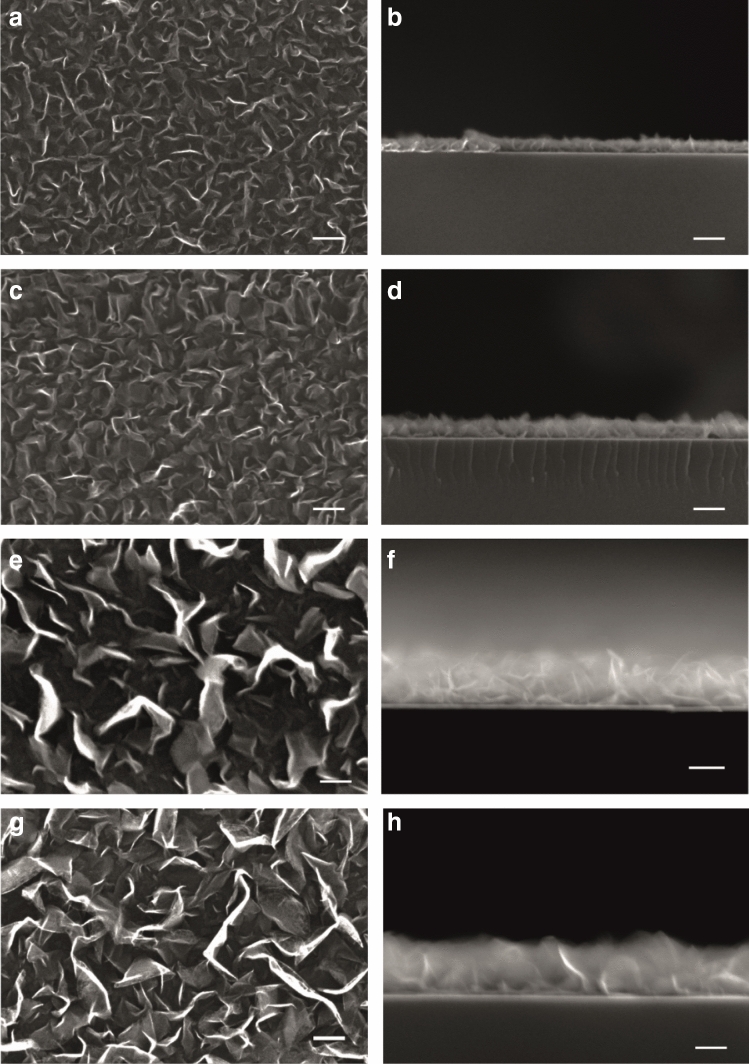
SEM images of CNW films grown on quartz substrates. Images in (**a**), (**c**), (**e**), and (**g**) show morphology (top-view) of CNW films, whereas images in (**b**), (**d**), (**f**), and (**h**) show a cross-section view of CNW films. Images in (**a**) and (**b**) correspond to the film growth time of 30 min; (**c**) and (**d**) correspond to the film growth time of 40 min; (**e**) and (**f**) correspond to the film growth time of 50 min; (**g**) and (**h**) correspond to the film growth time of 60 min. Scale bars in all images correspond to 100 nm.

Figure 1 shows SEM images of CNW films on quartz substrates with different growth time. As shown in the SEM images, the morphology of CNW films changes significantly with variations in the growth time (see top-view images). With the longer growth time, the length and thickness of the walls of CNWs increase and their density decreases. Also, the morphology of CNWs also changes as the growth time increases. For the growth time of 30 and 40 min, the obtained CNW films have a maze-like structure, whereas for 50 min and 60 min, CNW thin-films have a petal-like structure^27,35^. The height of the CNW films as well as the wall thickness of CNWs (vertically oriented graphene sheets) increase with the increase in the growth time. The thickness of the CNW films is around 60 nm for the shortest synthesis time (30 min), whereas for the longest growth time (60 min) it is around 190 nm. As for the intermediate growth times such as 40 and 50 min, the film thickness is around 85 nm and 160 nm, respectively (see Figure S1 in Supporting Information). The energy dispersive X-ray spectroscopy (EDS) analysis of the samples showed that the percentage of the carbon content (as opposed to silicon and oxygen coming from the substrate) increases with the increase in the growth time (see Figure S2 in Supporting Information). This also indicates the direct correlation between the film thickness and the film growth time.

**Figure 2:**
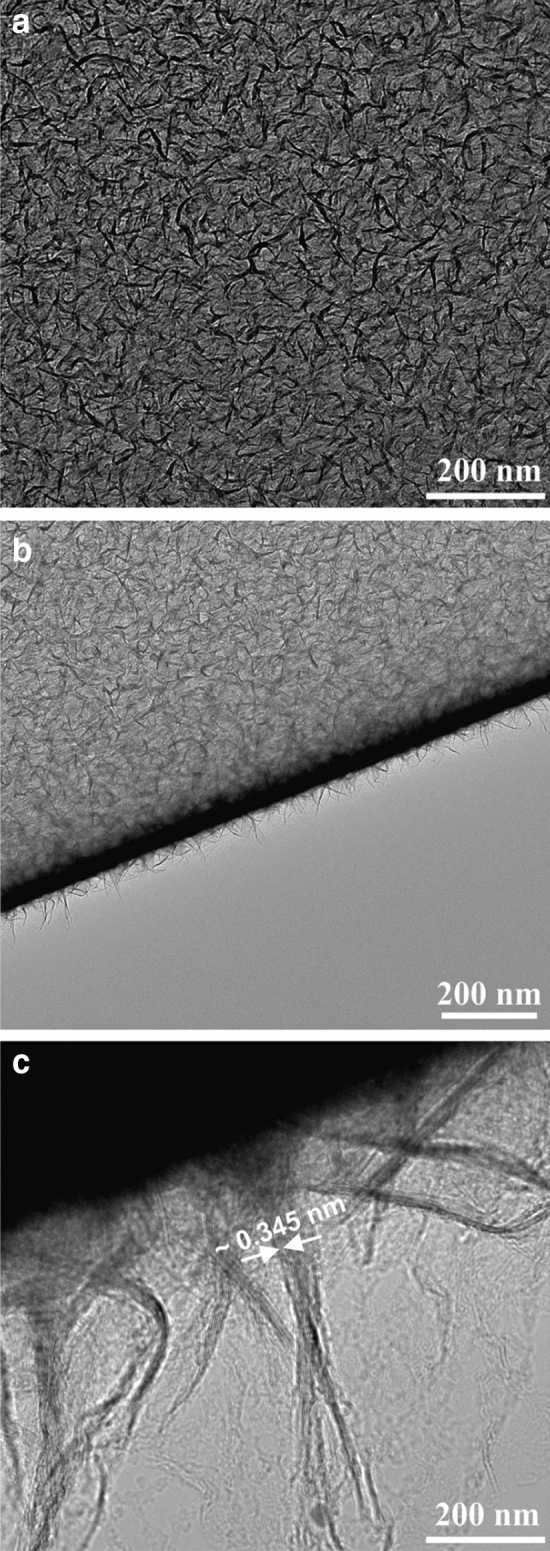
TEM images of a freestanding CNW film. (**a**) Top-view image of the CNW film. (**b**) Image of a coiled-up edge of the CNW film. (**c**) Higher magnification image of the coiled-up edge of the CNW film.

Figure 2 shows TEM images of a freestanding CNW film with the film growth time of 50 min. Figure 2a shows the top-view of the CNW film. The darker areas correspond to the vertically oriented CNWs, whereas the brighter areas correspond to a continuous CNW film oriented horizontally. Figure 2b shows a coiled-up edge of the CNW film. The image contrast arising from the coiled-up edge of the CNW film suggests that the CNW films are relatively transparent. A higher magnification image of the coiled-up edge of the CNW film (see Fig. 2c) indicates that the walls of CNWs consist of several layers of graphene sheets with the average distance between the layers being ~ 0.345 nm (see Figure S3 in Supporting Information). This is consistent with the d-spacing values for CNWs reported by other groups^35^.

**Figure 3:**
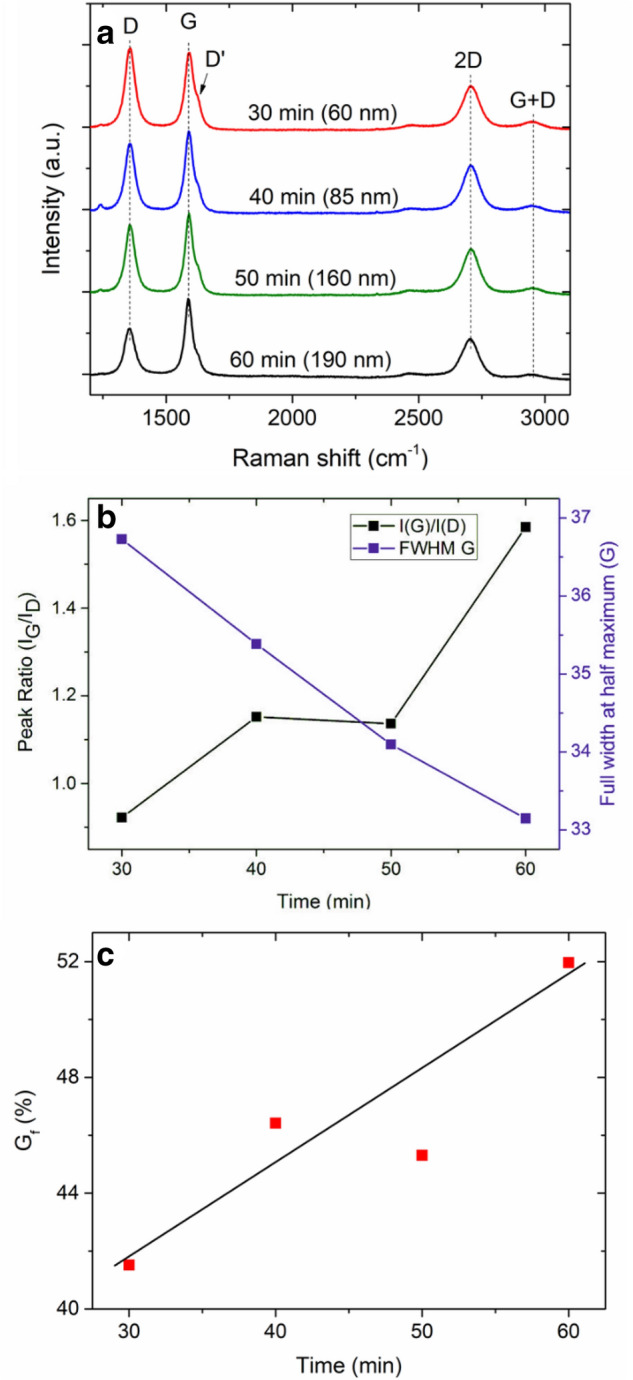
Raman spectroscopy analysis of the CNW films. (**a**) Spectra of the synthesized CNW films at various growth times (film thickness of the CNW films indicated in brackets). (**b**) Dependences of the ratio of the ${I}_{G}$ and ${I}_{D}$ peaks, and FWHM of the G peak on the film growth time. (**c**) Dependence of the estimated degree of graphitization in the CNW films on the film growth time.

Figure 3 shows the results of the structural analysis of the CNW films, obtained using Raman Spectroscopy. The Raman spectra of the samples displayed in Fig. 3a show a typical spectrum for CNWs with clear characteristic graphitic D, G, D′, G′ (2D), and G + D peaks^35^. The D band is associated with the defects in sp^2^ structures. The G peak is an inherent feature of graphitic materials. The D′ shoulder corresponds to the breaking of the symmetry of the finite-sized sp^2^ crystal and typical for graphene edges. The G′ (2D) peak is the second order of the D mode. The appearance of this peak indicates a long-range order in the structure. The G + D (D″) peak is a band that originates from the combination of G and D peaks^36^. Analysis of the Raman spectra shows that the ratio of the intensities of the G and D peaks (${I}_{G}/{I}_{D}$) increases with the increase in film growth time, which indicates the defect decrease in the structure of CNWs (Fig. 3b). The details of the Raman spectra analysis of the CNW films is in Supporting Information (see Table S1 and Figure S4). The relative amount of defects in the structure of the CNW films can be reflected from the full width at half maximum (FWHM) of the G peak. It is observed that the increase in film growth time results in decreasing FWHM as shown in Fig. 3b, indicating less defect density. This is an additional indication of the improved crystallinity of the graphite-like walls of the obtained CNW films.

It is possible to estimate the degree of graphitization in CNWs from the Raman spectrum using the following relation^37^:

1 $${G}_{f}=\frac{A (G)}{{\sum }_{900}^{1900}A}*100\%$$

Here, $A(G)$ is the area of the G peak, ${\sum }_{900}^{1900}A$ is the total spectrum area from 900 to 1900 nm measurement range. The degree of graphitization is a rather important structural characteristic, which indicates the nature of the carbon material between amorphous carbon (mix of sp^2^, sp^3^ and dangling bonds) and graphite (sp^2^ ordered structure). Figure 3c shows the dependence of the degree of graphitization on film growth time for the obtained CNW films. The value of $G(f)$ shows that the CNW films grown for 30 min have a degree of graphitization around 41%, whereas for the CNW film grown for 60 min, it is around 52%. Based on this and the results of the SEM, TEM, and Raman analysis data, it can be concluded that with an increase in the film growth time, the structure of CNW films undergoes a transformation from amorphous (turbostratic) carbon with small grain sizes to ordered graphite-like structure in the form of few-layer graphene flakes. We note that the Raman measurements shown in Fig. 3 were taken from the top surface of the CNW films and the recorded spectra are mostly originate from the top part of the CNW films. Hence, the analysis presented in Fig. 3 and Figure S4 (see Supporting Information) more relevant to the top part of the CNW films.

**Figure 4:**
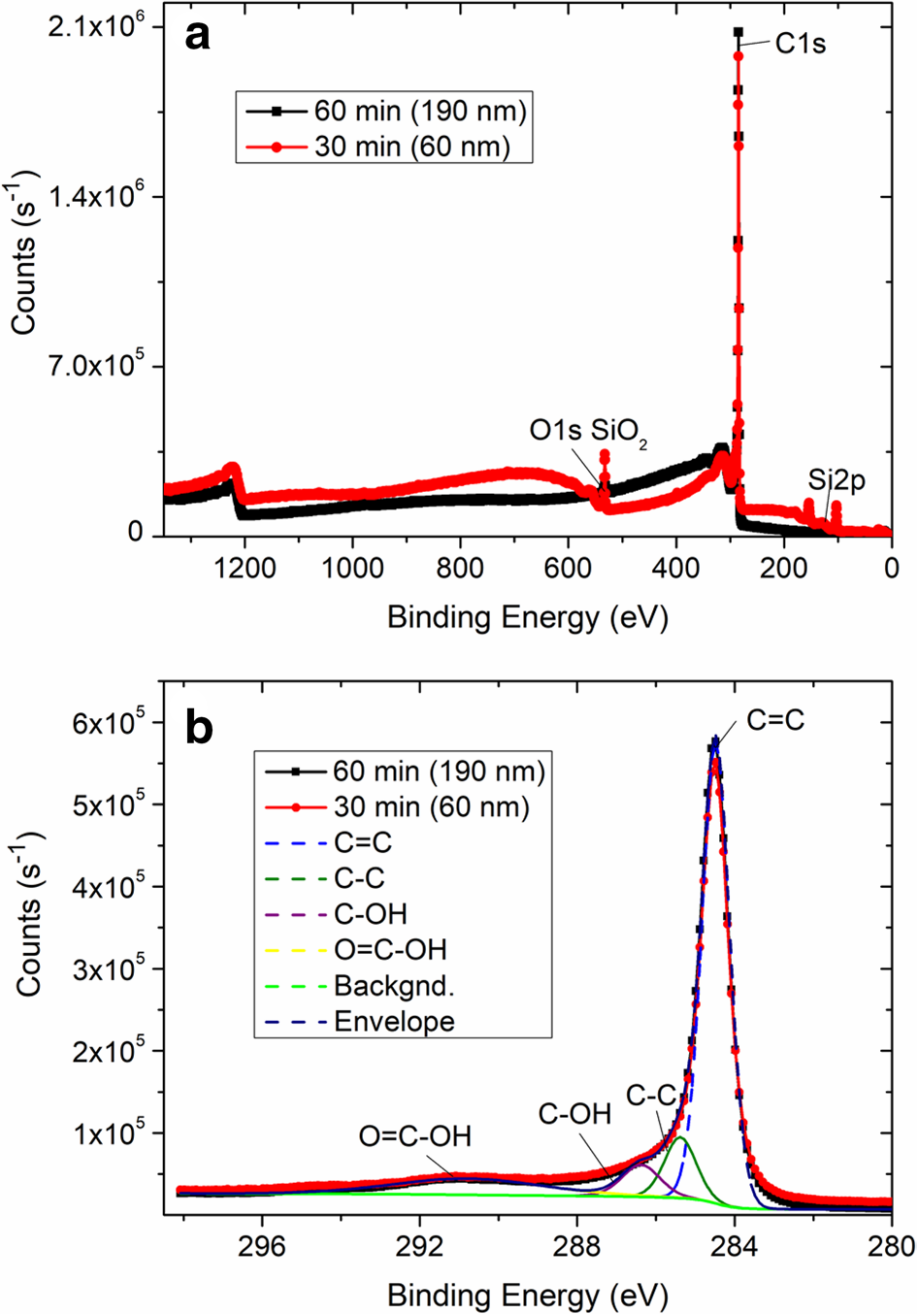
XPS analysis of the CNW films. (**a**) XPS spectra of the CNW films grown for 30 and 60 min (corresponding film thickness is indicated in brackets). (**b**) Deconvoluted C1s peak of the XPS spectra.

To analyze the chemical state of the obtained CNW films, we performed XPS measurements on the samples. Figure 4a shows an XPS spectrum of the CNW film grown for 30 and 60 min. The spectrum shows a pronounced intensive peak for carbon as well as peaks for silicon oxide (substrate material). It can be seen from the spectrum (Fig. 4a) that at a shorter synthesis time (30 min), the peaks of silicon oxide are more intense and pronounced compared to the results at 60 min, in which the carbon peak is higher than the peaks of silicon oxide. Figure 4b shows the C1s peak analyzed using a hybrid Gaussian-Lorentz approximation. Deconvoluted spectrum of the C1s peak shows that the peak consists of several peaks with the maxima at 284.5, 285.4, 286.4, and 290.7 eV. The peaks at 284.5 and 285.4 eV correspond to sp^2^ and sp^3^ hybridization, which is characteristic to the carbon double (C=C) and single (C–C) bonds, respectively^38^. The peaks at 286.4 and 290.7 eV indicate the existence of hydroxyl (C–OH) and carboxyl (O=C–OH) groups on CNWs, respectively^38^. This can be explained by the composition of the precursors (H_2_ and CH_4_) as well as the low-vacuum conditions during the synthesis process. Oxygen and hydrogen molecules can also be adsorbed by CNWs from the atmosphere after the synthesis due to the partly turbostratic nature of the structure with high specific surface area and chemically active dangling bonds.

The SEM and Raman spectroscopy analysis shown above indicate that the CNW films obtained at various film growth times differ morphologically and structurally. Shorter film growth times result in films with a relatively small-scale morphology and lower structural quality (e.g., defects), whereas longer film growth times result in films with a relatively large-scale morphology and better structural quality. In addition, the XPS analysis indicates the existence of hydroxyl and carboxyl functional groups on CNWs. These conditions may influence the electronic properties of CNW films.

**Figure 5:**
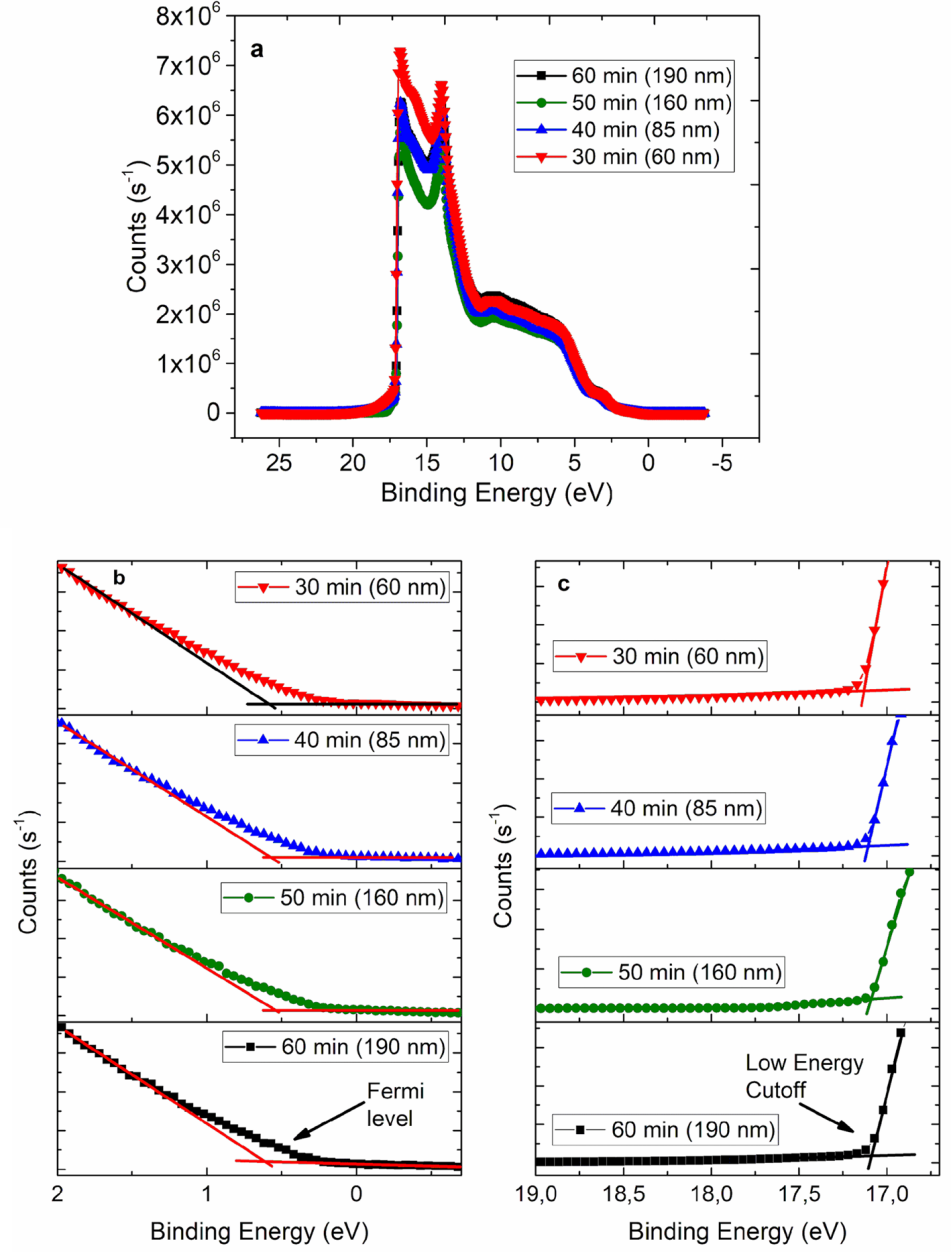
(**a**) UPS spectra of CNW films with different film growth times and film thickness (indicated in brackets). (**b**) Edge of the UPS spectra with the indicated Fermi level. (**c**) The UPS spectra of low energy cutoff with the onset highlighted in the graph to determine the work function.

Due to many applications of this material in various electronic devices, one of the important electronic parameters of CNWs is its work function^39^. Therefore, we performed UPS measurements on the CNW films with various film growth times in order to estimate their work function values. The UPS measurements were performed using He1α source (21.22 eV) and with a shift of − 10 eV (in order to calibrate the binding energy scale to the C1s core level state). Figure 5 shows UPS spectra of the CNW films grown for 30, 40, 50, and 60 min. The analysis of the UPS data indicates that there is a slight variation in the work function values for samples with different film growth times. The CNW film with the shortest growth time has a work function value of ~ 4.8 eV. When film growth time is increased, the work function values of the CNW films gradually drop. The CNW film with the longest synthesis time (60 min) has the work function value of ~ 4.7 eV. In graphitic materials, the values of work function may depend on the surface roughness, crystal orientation, and degree of graphitization^40,41^. An increase in the film growth time can lead to an increase in the size of graphite flakes and a decrease in the number of dangling bonds (at the edges of the walls). These factors can have an effect on the concentration of free charge carriers in the material and, thus, on the position of the Fermi level.

### Optical properties

**Figure 6:**
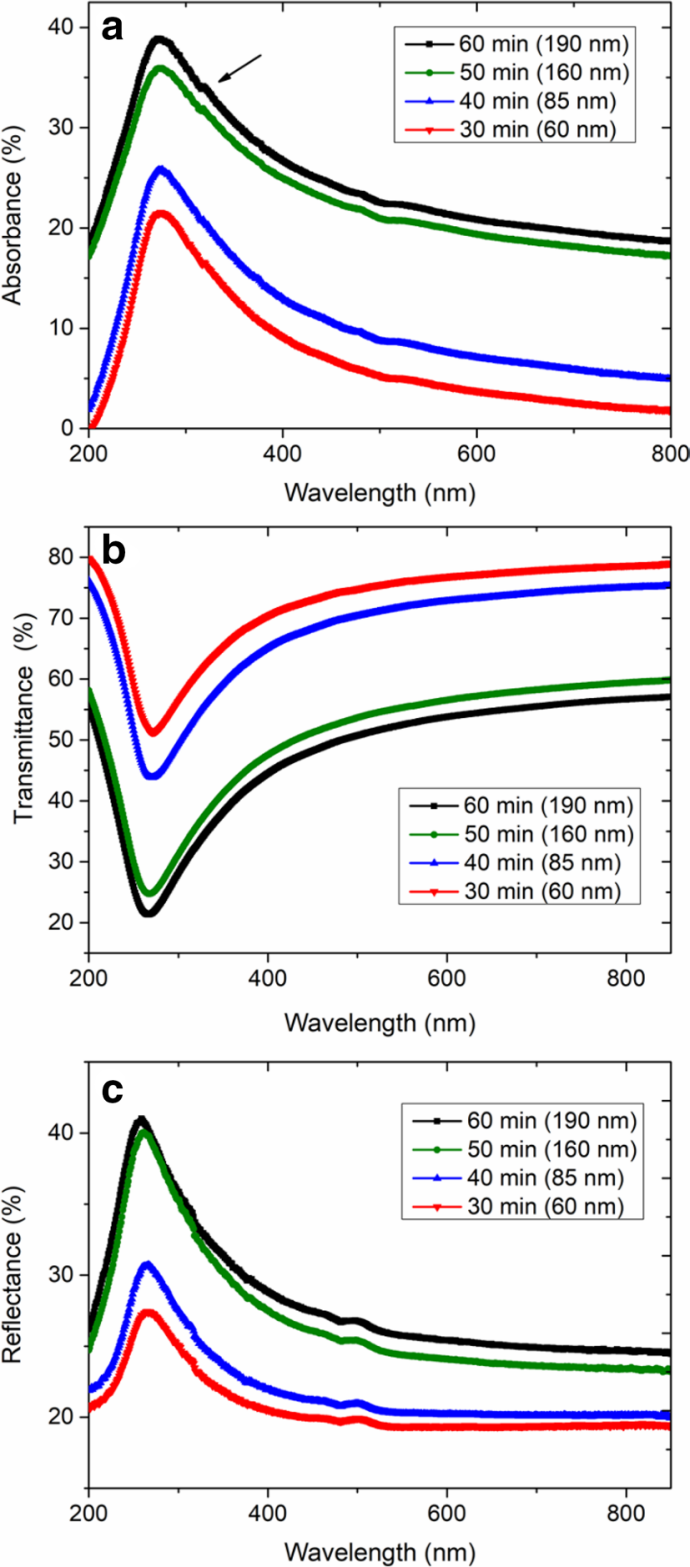
UV–Vis analysis of CNW films with different film growth times and film thickness (indicated in brackets). (**a**) Absorbance, (**b**) transmittance, and (**c**) reflectance spectra of the CNW films.

Since CNW films can be employed as a substitution for commonly used transparent conductive metal oxides such as fluorine-doped tin oxide or indium-doped tin oxide, it is useful to know the optical properties of CNW films. Figure 6 shows absorbance, reflectance, and transmittance spectra of the CNW films with different film growth times for the visible range. The shape of the measured absorbance, reflectance, and transmittance spectra for the CNW films is very similar to that of graphene sheets^42–45^. In the absorbance spectrum (Fig. 6a), all four samples show a distinct peak at ~ 250 nm. This is possibly associated with the π–π* transition caused by aromatic rings (C–C)^44,45^. A small shoulder at ~ 300 nm (the Fig. 6a is shown by an arrow) may originate from n–π* transitions due to carbonyl bonds (C=O)^45^. Figure 6b indicates that the transmittance of the CNW films decreases with the increase of film growth time. This is expected, since the thickness of the CNW films increase with longer film growth times (see SEM cross-section images in Fig. 1 and Figure S1 in Supporting Information). It is worth mentioning that the CNW film with the shortest synthesis time exhibits transmittance well above 70%, which is comparable to that of the thin films of commercially available conductive polymer materials such as PEDOT:PSS^46^. Figure 6c shows that the reflectance spectra of the CNW films increase with the increase in film growth time. This may originate from increased scattering due to large sized CNWs in the films with longer growth times. Indeed, the SEM top-view images shown in Fig. 1 depict that in the samples with a longer growth time, CNWs become large (in the order of several hundred nm), which increases the light scattering ability of the films.

### Electrical properties

**Figure 7:**
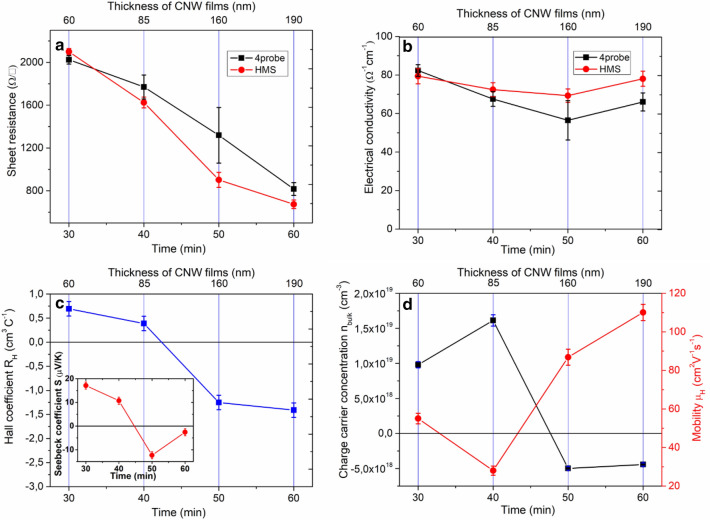
Electrical characteristics of the CNW films and their dependence on the synthesis time and film thickness. (**a**) Sheet resistance, (**b**) electrical conductivity, (**c**) Hall coefficient and Seebeck coefficient, and (**d**) charge carriers concentration (negative charge carriers concentration stands for electrons and the positive one for holes) and mobility.

Figure 7 shows how sheet resistance and conductivity of the CNW films vary with film growth time and thickness of the CNW films. The measurements are obtained using a Hall Effect measurement system and a four-point probing system. For Hall Effect analysis, 10 measurements are taken per data point with the Hall Effect measurement system, whereas 5 measurements are taken per data point with the four-point probing system. Figure 7a shows that the surface resistance of the CNW films decreases almost linearly with the increase in film growth time. The sheet resistance for the sample with the shortest growth time (30 min) is ~ 2000 Ohm/□, and for the longest growth time it is around 600 Ohm/□. Such a trend in variation of sheet resistance of the CNW films with the film growth time may originate from the increase in film thickness and the density (depending on the substrate) of CNWs with the longer growth time^47^. We note that there is a qualitative agreement between the values obtained with the Hall Effect measurements and four point probe measurements.

Figure 7b shows that the specific conductivity does not change linearly with film growth time. The conductivity values of the samples are roughly similar except for a small minimum observed for the sample grown for 50 min. Since the specific electrical conductivity is normalized by the film thickness, it represents the electrical properties of the film material itself, which should be in correlation with the structural and morphological characteristics of the CNW films obtained from the Raman spectroscopy measurements. However, the Hall Effect and the four-probe techniques measure the specific conductivity of the samples in the lateral direction (horizontal to the substrate surface). This means that the structural characteristics of the bottom part of the CNW films (close to the substrate) will have a dominant effect on the specific conductivity of the samples. However, our observations on the structural properties of the CNW films obtained from the Raman measurements (see Fig. 3) are more relevant to the top part of the CNW films, and it is difficult to make any definite conclusions on the structural properties of the bottom part of the CNW films from these measurements. The more or less similar values of the specific conductivity in all samples regardless of the growth time indicate that the bottom part of the CNWs films in all samples might have a similar film thickness and electrical properties. This result can be rationalized if we consider the growth mechanism of CNW films^2,48–50^. After the nucleation period, the isolated nanosheets on the substrate with random orientation would start to grow. Eventually, the neighboring nanosheets will meet each other. With further increase in growth time, semicircular nanographene would spread preferably, and growth of inclined sheets would be blocked by the vertical sheets grown faster, resulting in the formation of typical CNWs. The longer growth time results in film thickness increase and some structural improvements in the top part of the CNW film, however the bottom part of the CNW film most likely remain the same. Such a scenario would be consistent with the results of electrical conductivity measurements shown in Fig. 7b.

Figure 7c shows the measured Hall coefficients of the CNW films under investigation. The Hall coefficients (${R}_{H}$) for different film growth time show that the CNW films, grown for 30 and 40 min, exhibit p-type conductivity, whereas the CNW films grown for 50 and 60 min exhibit n-type conductivity. It is known that graphene-like structures are an environmentally sensitive materials which can reversibly change their type of conductivity based (depending) on the conditions of chemical doping^51^. Therefore, the observed switch of the type of conductivity of the CNW films, obtained during different growth times, can be caused by the difference in the surface area and the amount of adsorbed heterogeneous atoms and molecules from the CVD chamber and laboratory environments. However, it is necessary to mention that the sign of the measured Hall coefficient is known to be compromised by the dominant hopping mechanism of charge transport in disordered mediums^7,38,52–54^. Taking into account the relatively low degree of crystallinity in the studied CNW films, there is a possibility that the measured sign of the Hall Effect does not correctly correlate with the type of majority charge carriers. Thus, the measurement of the thermoelectric voltage and corresponding Seebeck coefficients of the CNW films was carried out as an additional experiment to test the conductivity type of the CNW films (Figure S4). The sign of the Seebeck coefficient is not compromised by the hopping charge transport and always correctly reveals the dominant type of conductivity^54^. It is seen from the inset in Fig. 7c that the measured Seebeck coefficient changes its sign from positive (p-type) to negative (n-type) with the increase of the growth time. Thus, the thermoelectrical characterization independently confirms the data obtained by the Hall effect method^55^.

Figure 7d shows the dependence of charge carrier concentration (${n}_{bulk}$) and their mobility (${\mu }_{H}$) in the CNW films on the film growth time and obtained by the Hall Effect method. Apart from a small minimum for the film growth time of 40 min, carrier mobility seems to increase with the increase in film growth time. The increase in carrier mobility values with the increase in film growth time might originate from the reduced defect density in the samples (especially in the lower part of CNW films) with longer synthesis time. This may not be deduced directly from the Raman spectroscopy measurements shown in Fig. 3 since these findings are more relevant to the top part of the CNW films. However, it is reasonable to assume that the reduction in the defect density in the horizontally oriented graphite nanosheets at the base of CNWs is somewhat proportional to the growth time^38,50,56,57^, meaning that under the synthesis conditions (high temperatures and presence of feedstock material), the defects in the horizontally oriented graphite nanosheets at the base of CNWs are also slowly healed/removed with prolonged growth time. We note that the carrier mobility value of the CNW film grown for 60 min is only a factor of ~ 2 higher than that of grown for 30 min. The significance of this, of course, depends on the application purposes of CNW films. However, further studies that are more comprehensive (e.g., time resolved microwave conductivity measurements, field-effect mobility measurements, etc.) are required to shed light on the dependence of carrier mobility in CNW films on growth time.

## Conclusions

In conclusion, the synthesis of CNW films on quartz substrates using the ICP-PECVD method is demonstrated. The obtained CNW films consist of vertically oriented few-layer graphene sheets with heights varying from 60 to 190 nm. Depending on the film growth time, the morphology of the CNW films changes from a maze-like structure (30–40 min) to a petal-like structure (50–60 min). The structural, electronic and optical properties of the samples are characterized with various analytical techniques. The analysis of the Raman spectra of the samples showed that the obtained materials are CNWs with corresponding peaks at the respective Raman shift. The ratio of the ${I}_{G}/{I}_{D}$ Raman peaks increases with the increase in film growth time. The analysis of FWHM of the G Raman peak shows a narrowing of the G peak from 37.84 to 33.27 cm^−1^, and the calculation of the degree of graphitization changes from 41% (30 min) to 52% (60 min). These findings indicate that the structural properties of the CNW films (the top part of the film) improve with the increase in growth time. Also, the influence of the CNWs morphology on various optical, structural, and electrical properties of the material is revealed. In particular, the Hall and Seebeck effect measurements of the samples reveal that CNW films with a maze-like morphology (film growth time 30 and 40 min) exhibit p-type semiconducting properties, whereas CNW films with a petal-like morphology (film growth time 50 and 60 min) exhibit n-type of conductivity. The observed change in the type of conductivity deserves additional detailed investigations due to its potential to broaden the set of suitable semiconductor materials for the fabrication of CNW/semiconductor Schottky-type heterojunction photodiodes or gas sensors^58,59^.

## Supplementary Information

Supplementary Information.
