# Supplementary material for: Physical properties of carbon nanowalls synthesized by the ICP-PECVD method vs. the growth time
Source: Sci Rep. 2021 Sep 29;11:19287. doi: 10.1038/s41598-021-97997-8 (PMC8481469; doi:10.1038/s41598-021-97997-8)
Supplement: Supplementary file 1 — Supplementary Information. [file 41598_2021_97997_MOESM1_ESM.docx]

**Supporting Information**

**Physical Properties of Carbon Nanowalls Synthesized by the ICP-PECVD method vs. the Growth Time**

Yerassyl Yerlanuly^1,2,3^, Rakhymzhan Zhumadilov^1^, Renata Nemkayeva^2,4^, Berik Uzakbaiuly^5^, Almaz R. Beisenbayev^3,6^, Zhumabay Bakenov^5,6^, Tlekkabul Ramazanov^4^, Maratbek Gabdullin^2,4^, Annie Ng^7^, Viktor V. Brus^3*^ and Askhat N. Jumabekov^3*^

^1^ Laboratory of Engineering Profile, Al-Farabi Kazakh National University, Almaty 050040, Kazakhstan

^2^ Kazakh-British Technical University, Almaty 050000, Kazakhstan

^3^ Department of Physics, Nazarbayev University, Nur-Sultan 010000, Kazakhstan

^4^ National Nanotechnology Laboratory Open Type, Al-Farabi Kazakh National University, Almaty 050040, Kazakhstan

^5^ National Laboratory Astana, Nur-Sultan 010000, Kazakhstan

^6^ Department of Chemical and Materials Engineering, Nazarbayev University, Nur-Sultan 010000, Kazakhstan

^7^ Department of Electrical and Computer Engineering, Nazarbayev University, Nur-Sultan 010000, Kazakhstan

*** Corresponding Authors**

Viktor V. Brus. Email: viktor.brus@nu.edu.kz

Askhat N. Jumabekov. Email: askhat.jumabekov@nu.edu.kz

**Sample preparation for TEM analysis of CNWs.**

Preparation of CNWs samples for TEM studies are carried out as follows. Scratches were made on the obtained CNWs samples on a quartz substrate down to the quartz surface itself, and distilled water drops were applied to the place where the scratch had formed. As a result, the CNWs film floated on to the surface of the water, then this film was caught on a copper grid for TEM measurements.





**Figure S1**. Dependence of the thickness of the CNW films on the synthesis time


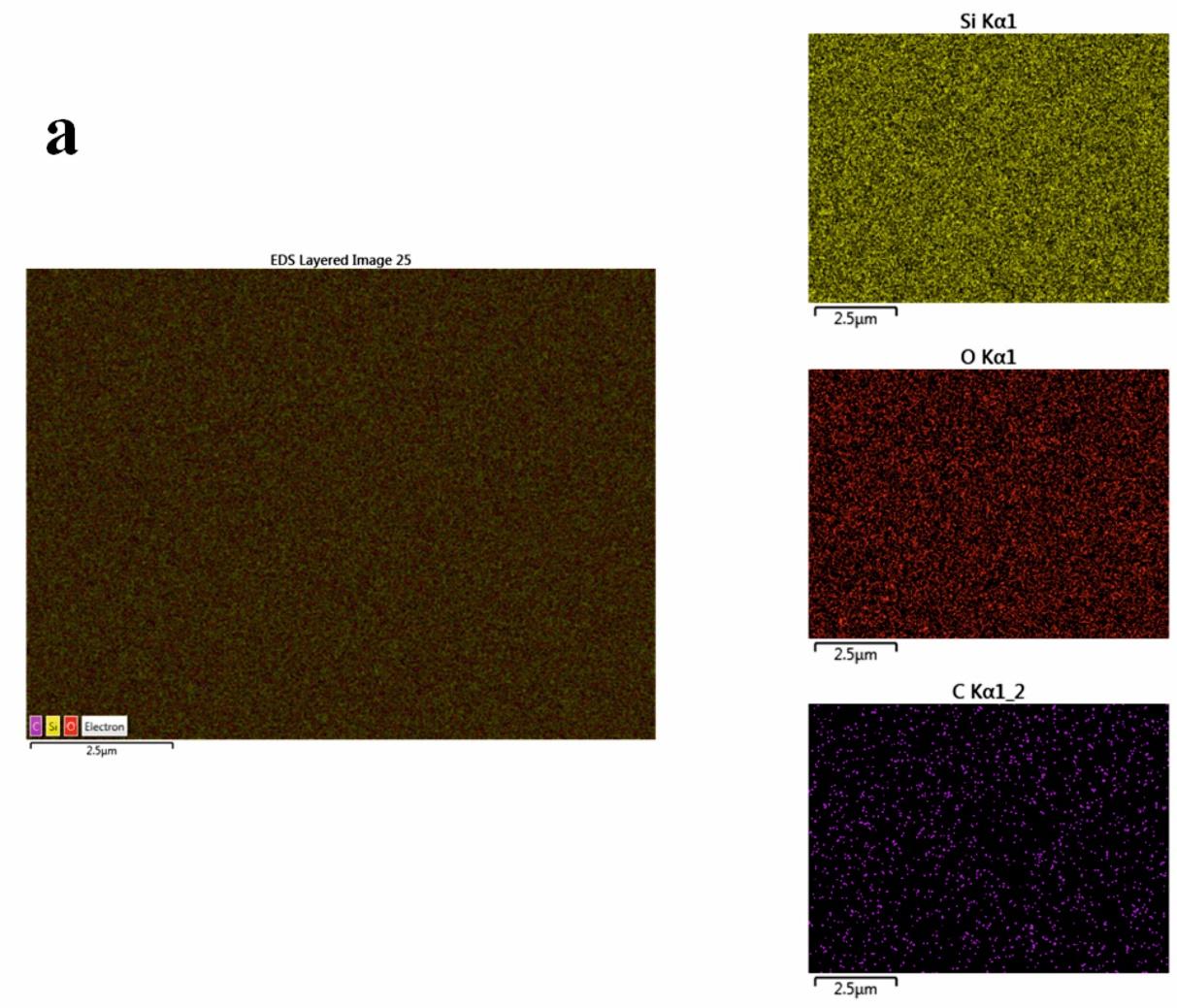


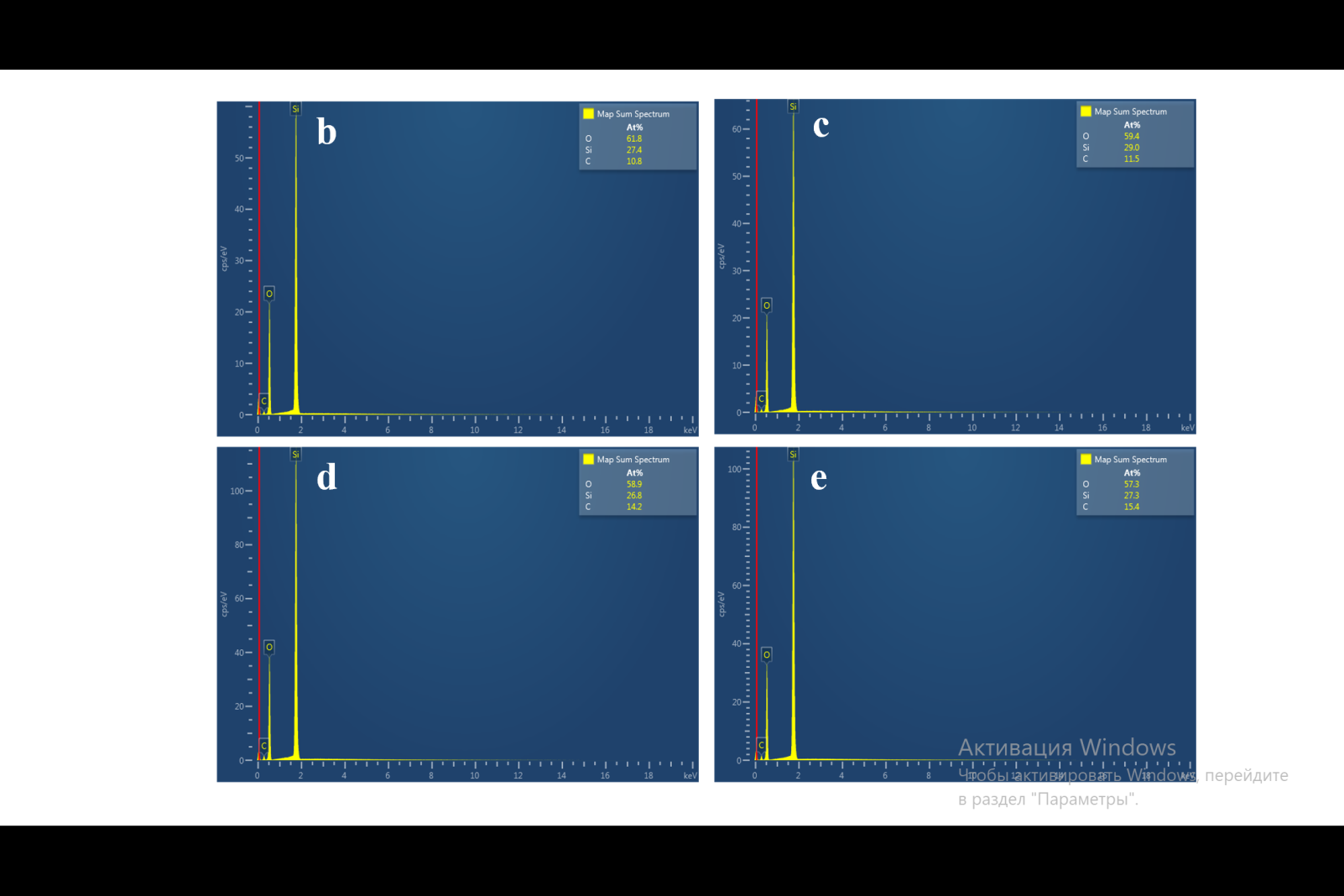


**Figure S2**. EDS analysis of CNW thin-films grown on quartz substrates. a) EDS mapping, b) corresponds to the film growth time of 30 min; c) corresponds to the film growth time of 40 min; d) corresponds to the film growth time of 50 min; e) corresponds to the film growth time of 60 min.


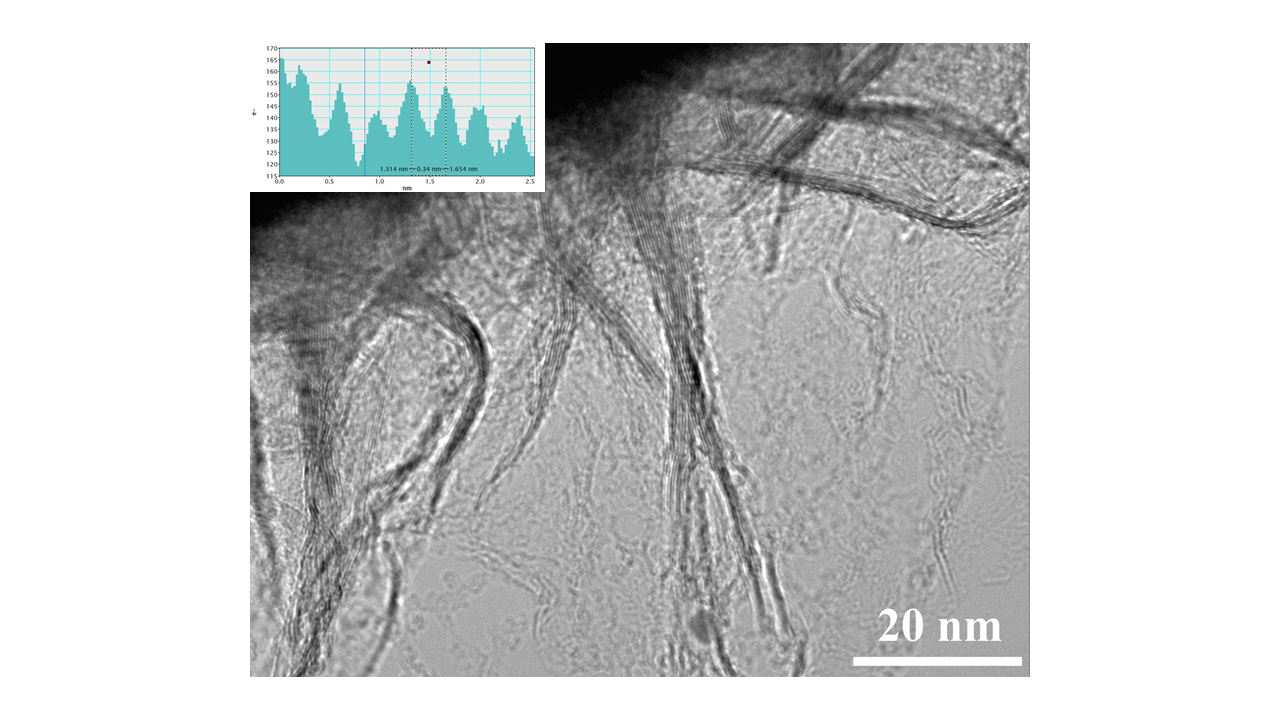


**Figure S3**. Higher magnification TEM image of a coiled-up edge of the CNW thin-film. The average distance between the graphite sheets is 0.345 nm as shown in the analysis (inset figure in the upper left corner) of the image.

**Table S1**. Raman spectroscopy analysis data.

|  | 30 min | 40 min | 50 min | 60 min |
| --- | --- | --- | --- | --- |
| G peak position | 1591.37 | 1590.47 | 1590.60 | 1588.84 |
| D peak position | 1357.09 | 1357.29 | 1357.85 | 1355.89 |
| D' peak position | 1624.84 | 1625.68 | 1625.97 | 1625.00 |
| FWHM G | 37.84 | 36.30 | 34.96 | 33.27 |
| FWHM D | 45.37 | 45.22 | 44.33 | 43.60 |
| I(G)/I(D) | 0.92 | 1.15 | 1.13 | 1.59 |
| I(G)/I(D') | 4.96 | 6.42 | 5.71 | 8.39 |
| I(G)/I(2D) | 1.74 | 1.70 | 1.73 | 1.92 |
| I(G)/I(D+G) | 10.51 | 12.75 | 13.09 | 19.51 |


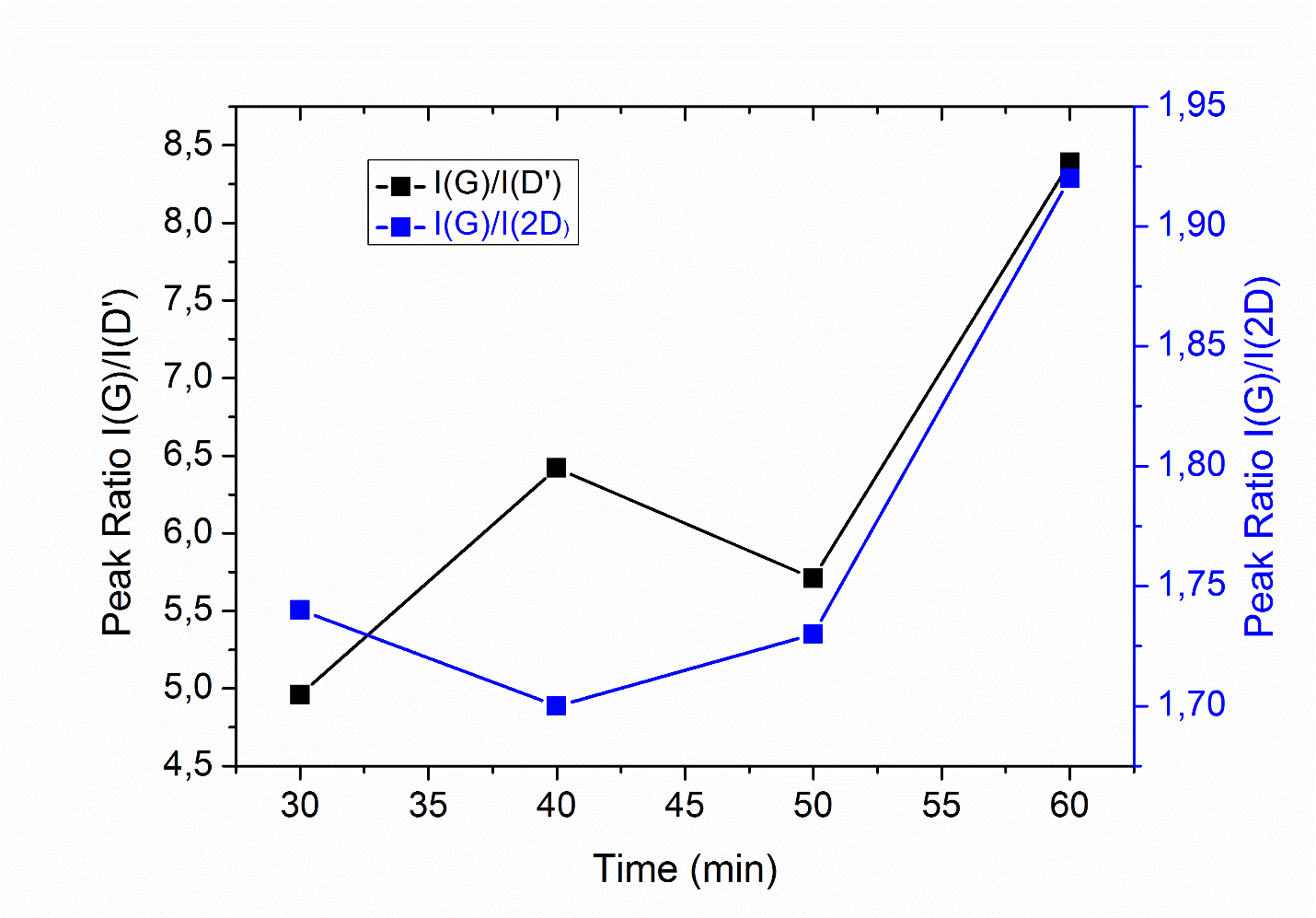


**Figure S4**. I(G)/I(D’) and I(G)/I(2D’) peak intensity ratios for Raman spectra. The I(G)/I(D’) peak intensity ratio indicates the level of disorder in graphitic structures. The relative intensity of the 2D peak is usually used for the evaluation of the thickness of graphene (briefly, the higher 2D peak the lower the number of graphene layers). However, the intensity of this band is also affected by the level of defects (and long-range order). Taking into account the observed dependence of morphology on the synthesis time (SEM images), it can be concluded that the increase of I(G)/I(2D) ratio is due to the increase in thickness of the walls (FLG flakes). The appearance of D’ peak in Raman spectrum of graphitic materials usually indicates the presence of defects, particularly vacancies in sp^2^ structure.


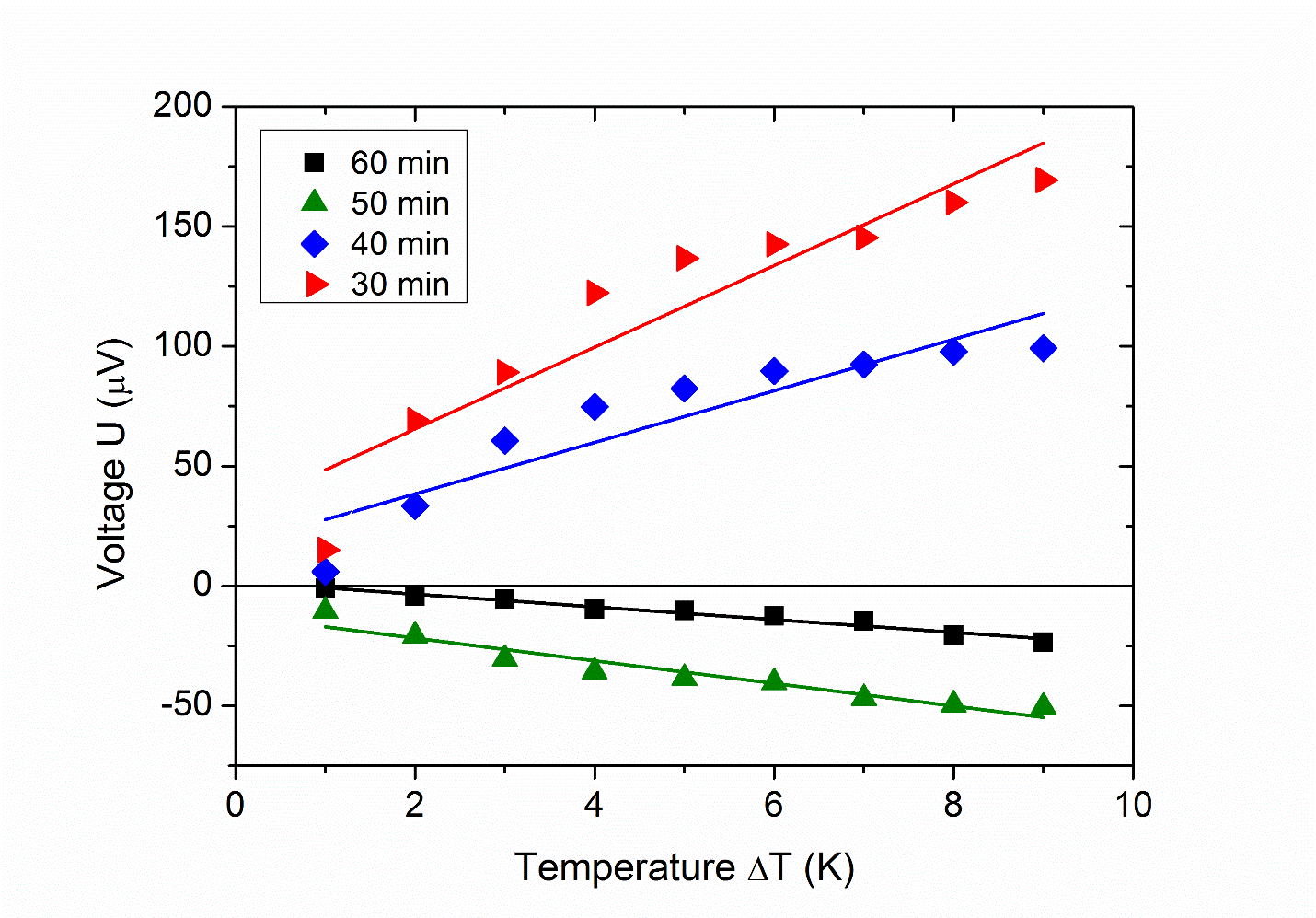
 **Figure S5**. Thermoelectric voltage versus temperature.
